# Author Correction: Lung endothelial cell antigen cross-presentation to CD8^+^T cells drives malaria-associated lung injury

**DOI:** 10.1038/s41467-019-13025-4

**Published:** 2019-11-04

**Authors:** Carla Claser, Samantha Yee Teng Nguee, Akhila Balachander, Shanshan Wu Howland, Etienne Becht, Bavani Gunasegaran, Siddesh V. Hartimath, Audrey W. Q. Lee, Jacqueline Theng Theng Ho, Chee Bing Ong, Evan W. Newell, Julian Goggi, Lai Guan Ng, Laurent Renia

**Affiliations:** 1 Singapore Immunology Network (SIgN), A*STAR, 8A Biomedical Grove, Level 3 & 4 Immunos Building, Singapore, 138648 Singapore; 2 Department of Microbiology and Immunology, Yong Loo Lin School of Medicine, National University of Singapore, 5 Science Drive 2 Blk MD4, Level 3, Singapore, 117545 Singapore; 3 Isotopic Molecular Imaging Laboratory, Singapore Bioimaging Consortium (SBIC), A*STAR, 11 Biopolis Way, #02-02 Helios, Singapore, 138667 Singapore; 4 Histolopathology/Advanced Molecular Pathology Lab, Institute of Molecular and Cell Biology (IMCB), A*STAR, 61 Biopolis Drive, Level 6 Proteos Building, Singapore, 138673 Singapore

**Keywords:** Cellular immunity, Antigen-presenting cells, Malaria, Parasite host response

Correction to: *Nature Communications* 10.1038/s41467-019-12017-8, published online 18 September 2019.

The original version of this Article contained an error in one of the dot plots in Fig. 2d. The percentage of Pb1^+^GrzB^+^ cells in Pb*Aluc*-infected mice was incorrectly given as ‘0’ and should have been ‘69.6’. This error is now corrected in both the PDF and HTML versions of the Article.

