# Supplementary material for: Lung endothelial cell antigen cross-presentation to CD8+T cells drives malaria-associated lung injury
Source: Nat Commun. 2019 Sep 18;10:4241. doi: 10.1038/s41467-019-12017-8 (PMC6751193; doi:10.1038/s41467-019-12017-8)
Supplement: Supplementary file 1 — Supplementary Information [file 41467_2019_12017_MOESM1_ESM.pdf]

## Supplemental Material

# Lung endothelial cell antigen cross-presentation to CD8$^{+}$T cells drives malaria-associated lung injury

Claser and Nguee et al.

| Supplementary Table 1: Cell Number and percentage of immune cells subtypes present in the lungs of PbA*luc*-infected mice on 7 dpi | | | | | |
|---|---|---|---|---|---|
| | **Cell number** | | **Percentage (%)** | | |
| | N (n=9) | PbA (n=11) | N | PbA | *P* Value* |
| **Absolute total cell count** | 6120000 ± 2369240 | 5783636 ± 2390512 | | | NS |
| **CD4** | 127635 ± 72433 | 175260 ± 78096 | 16.35 | 16.42 | NS |
| **CD8** | 83694 ± 47860 | 184225 ±107155 | 10.72 | 17.26 | b |
| **NK** | 152189 ± 109855 | 175505 ± 94105 | 19.49 | 16.44 | NS |
| **Monocytes** | 60561 ± 30084 | 131079 ± 111770 | 7.76 | 12.28 | NS |
| **Neutrophils** | 290993 ± 249427 | 140054 ± 128675 | 37.27 | 13.12 | NS |
| **cDC** | 5889 ± 4292 | 1214 ± 1886 | 0.75 | 0.11 | c |
| **pDC** | 4202± 2339 | 555.9 ± 549.8 | 0.54 | 0.05 | d |
| **Mono derived DC** | 9016 ±14568 | 27286 ± 19980 | 1.15 | 2.56 | b |
| **Macrophages** | 18150 ± 14590 | 4891 ±6493 | 2.32 | 0.46 | b |
| **Interst Macro** | 2868 ± 3846 | 4079 ± 3714 | 0.37 | 0.38 | NS |
| **MDM** | 13332 ± 17104 | 194144 ± 141333 | 1.71 | 18.19 | d |
| **Alveolar macro** | 12322 ± 8326 | 29297 ± 47219 | 1.58 | 2.74 | NS |
| | | | | | |
| ***Definition of abbreviations***: **N = naïve; cDC = conventional DC; pDC = plasmocytoid DC; MDM = mono derived macrophages; NS = not significant** | | | | | |
| ***Significance level of naïve versus PbAluc 7 dpi. Cell numbers are indicated in the right-hand end column. Results compiled from 3 independent experiments. Significance levels are indicated as follows: $^{a}$*P*<0.05; $^{b}$*P*<0.01; $^{c}$P<0.001; $^{d}$*P*<0.0001 Unpaired-t test or Mann-Whitney test was used** | | | | | |

| Supplementary Table 2: Antibodies used for Flow Cytometry | | | | | |
|---|---|---|---|---|---|
| **Antibody** | **Clone** | **Fluorochrome** | **Dilution** | **Catalog number** | **Supplier** |
| ***Leukocytes*** | | | | | |
| **CD45** | 30-F11 | APC-Cy7 | 1:250 | 557659 | BD Pharmingen |
| **CD3e** | 145-2c11 | BV421 | 1:100 | 100336 | Biolegend |
| **CD8** | 53-6.7 | BV605 | 1:200 | 100744 | Biolegend |
| **CD4** | RM4-5 | PE-Cy7 | 1:200 | 25-0042-82 | eBioscience |
| **NK1.1** | Pk136 | APC | 1:100 | 17-5941-82 | eBioscience |
| **CXCR3** | CXCR3-173 | PerCP-Cy5.5 | 1:100 | 45-1831-82 | eBioscience |
| **CD11a (LFA-1)** | M17/4 | FITC | 1:250 | 11-0111-82 | eBioscience |
| **$IA^b/IE^b$ (MHC II)** | M5/114.15.2 | Alexa700 | 1:200 | 56-5321-82 | eBioscience |
| **CD11b** | M1/70 | BV650 | 1:200 | 101239 | Biolegend |
| **CD11c** | N418 | PE-Cy7 | 1:200 | 25-0114-82 | eBioscience |
| **CD24** | M1/69 | Pacific blue | 1:200 | 48-0242-82 | eBioscience |
| **CD64** | X54-5/7.1.1 | PE | 1:100 | 558455 | BD Pharmingen |
| **Ly6C** | HK1.4 | PerCP-Cy5.5 | 1:200 | 45-5932-82 | eBioscience |
| **F4/80** | BM8 | Biotin | 1:50 | 123106 | Biolegend |
| **FceR1 alpha** | MAR1 | APC | 1:50 | 17-5898-82 | eBioscience |
| **BST2** | ebio927 | FITC | 1:200 | 11-3172-82 | eBioscience |
| ***Intracellular staining*** | | | | | |
| **IFN-γ** | XMG1.2 | PerCP-Cy5.5 | 1:50 | 45-7311-82 | eBioscience |
| **Granzyme B** | NGZB | PE-Cy7 | 1:100 | 25-8898-82 | eBioscience |
| ***Endothelial cells*** | | | | | |
| **CD31** | 390 | PE-Cy7 | 1:50 | 102418 | Biolegend |
| **CD144 (VE-Cad)** | BV13 | APC | 1:100 | 17-1441-82 | eBioscience |
| **CD34** | RAM34 | Alexa700 | 1:100 | 56-0341-82 | eBioscience |
| **L-selectin (CD62L)** | MEL-14 | efluor450 | 1:100 | 48-0621-82 | eBioscience |
| **$H\text{-}2D^b$ (MHC I)** | KH95 | FITC | 1:200 | 111505 | Biolegend |
| **$IA^b/IE^b$ (MHC II)** | M5/114.15.2 | efluor450 | 1:200 | 48-5321-80 | eBioscience |

| Supplementary Table 3: Antibodies used for CyTOF | | |
|---|---|---|
| **Metal** | **Antibodies** | **Clone** |
| Y-89 | CD45 | Fluidigm |
| Qdot 655 112/114 | CD19 | Fluidigm |
| In-115 | CD90 | T24B1 |
| Ce-140 | Ly6C | HK1.4 |
| Pr-141 | CD43 | S11 |
| Nd-142 | TCR-beta | H57-597 |
| Nd-143 | CD49d | PS/2 |
| Nd-144 | CD38 | 90 |
| Nd-145 | GITR | DTA-1 |
| Nd-146 | CD8a | 53-6.7 |
| Sm-147 | OX40 | OX-86 |
| Nd-148 | CD69 | H1.2F3 |
| Sm-149 | CD4 | H129.19 |
| Nd-150 | ICOS | C398.4A |
| Eu-151 | CD62L | MEL14 |
| Sm-152 | CD127 | A7R34 |
| Eu-153 | CD49b | DX5 |
| Sm-154 | CXCR3 | CXCR3-173 |
| Gd-156 | CD27 | LG.7F9 |
| Gd-157 | Ror-gamma T | 4G419 |
| Gd-158 | KLRG-1 | 2F1 |
| Tb-159 | 2B4 | m2B4 (B6)458.1 |
| Gd-160 | Ki67 | 11F6 |
| Dy-161 | CD25 | PC61 |
| Dy-162 | PD-1 | 29F11.1.112 |
| Dy-63 | CD122 APC | TM-β1 |
| Dy-164 | CD39 | 5F2 |
| Ho-165 | Eomes | 7C9B03 |
| Er-166 | CD160 | 7H1 |
| Er-167 | CD150 PE | TC15-12F12.2 |
| Er-168 | CD272 | 6A6 |
| Tm-169 | CD 103 | 2 E7 |
| Er-170 | TIM-3 | B8.2C12 |
| Yb-171 | CD73 | Ty/23 |
| Yb-172 | Sca-1 | D7 |
| Yb-173 | FOXP3 | MF-14 |
| Yb-174 | CD24 | M1/69 |
| Lu-175 | CD44 | IM7 |
| Yb-176 | Lag-3 | C9B7W |
| Ir-191/193 | DNA | |
| Pt-195 | CisPlatin live/dead | |

| Supplementary Table 4: Cell Number and percentage of immune cells subtypes present in the lungs of PbA*luc*-infected mice (CTR) and PbA*luc*-infected mice depleted of $CD8^+$ T cells (αCD8β) at 6dpi | | | |
|---|---|---|---|
| | **Cell number** | | |
| | CTR (n=9) | αCD8β (n=12) | *P* Value* |
| **Absolute total cell count** | 6450000 ± 2314768 | 6153750 ± 1540447 | NS |
| **CD4** | 165865 ± 71234 | 185526 ± 63522 | NS |
| **CD8** | 274301 ± 78473 | 43211 ± 25551 | d |
| **NK** | 177186 ± 80728 | 244917 ± 72843 | NS |
| **Monocytes** | 72326 ± 62744 | 180454 ± 93985 | b |
| **Neutrophils** | 180216 ± 228737 | 170227 ± 133824 | NS |
| **cDC** | 1889 ± 924.6 | 945.1 ± 409.6 | b |
| **Mono derived DC** | 13074 ± 9304 | 18953 ± 15126 | NS |
| **Macrophages** | 522.3 ± 416.3 | 1596 ± 635.3 | c |
| **Interst Macro** | 2968 ± 2226 | 3971 ± 2841 | NS |
| **MDM** | 112941 ± 89194 | 232297 ± 80193 | b |
| **Alveolar macro** | 2060 ± 1877 | 2448 ± 1450 | NS |
| | | | |

***Definition of abbreviations*: N = naïve; cDC = conventional DC; MDM = mono derived macrophages; NS = not significant**

***Significance level of CTR versus αCD8β on 7 dpi. Cell numbers are indicated in the right-hand end column. Significance levels are indicated as follows: $^{b}P$<0.01; $^{c}P$<0.001; $^{d}P$<0.0001. Mann-Whitney test was used.**

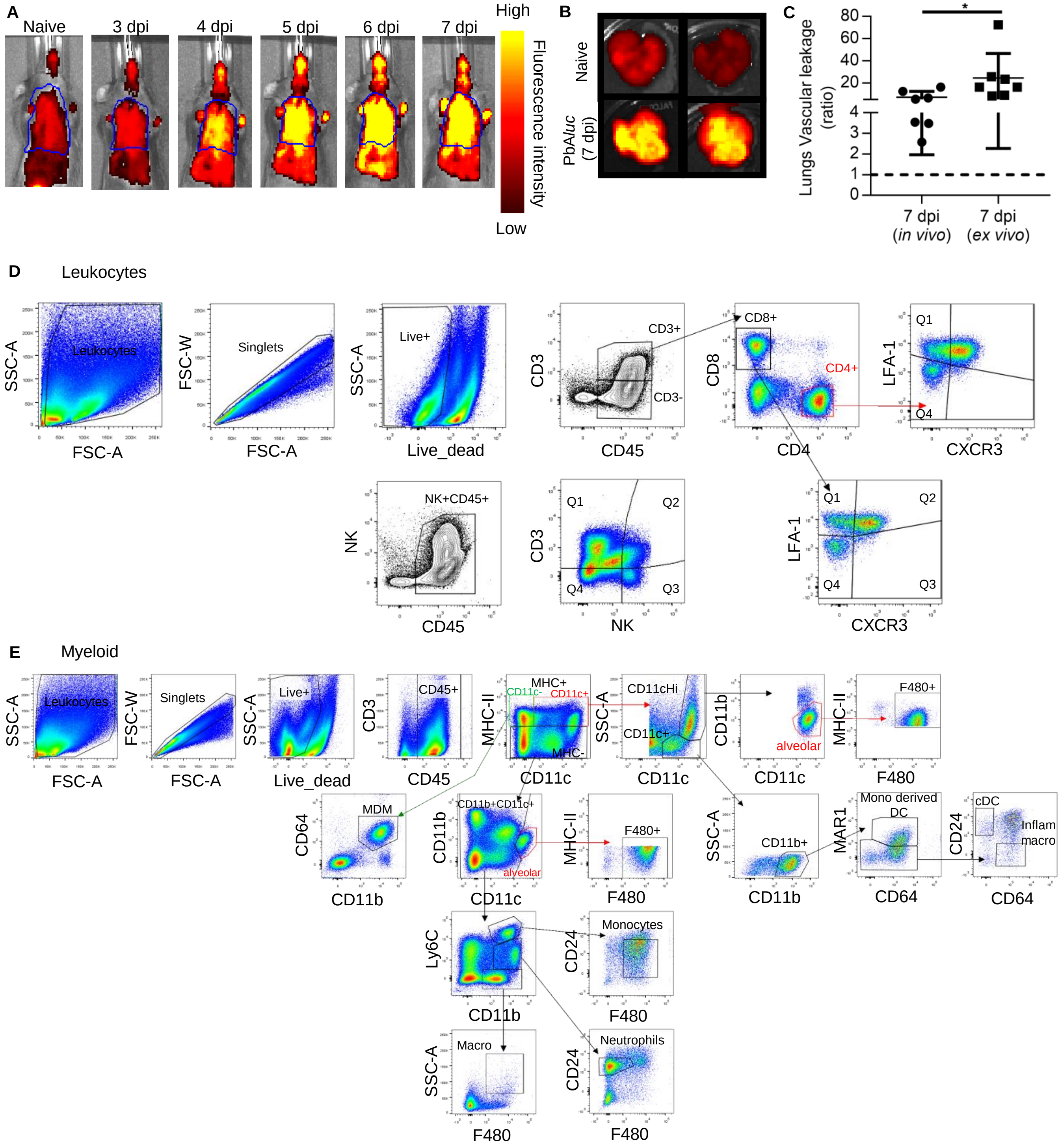


**Supplementary Figure 1.** Representative images of (A) *in vivo* and (B) *ex vivo* vascular leakiness in the lungs of PbA*luc*-infected mice. The blue line outlines the lungs region of interest (ROI) used to quantify the Tracer-653 dye signal intensity. (C) Comparison between *in vivo* and *ex vivo* vascular leakiness in the lungs of PbA*luc*-infected mice at 7 dpi. The black dashed line at y=1 in (C) represents the ratio of the tracer reading from naïve C57BL/6 mice (n=3). The data represent the mean ± SD; *p<0.05 by Mann-Whitney test. Gating strategy used to analyze flow cytometry data of (D) leukocytes and (E) myeloids sequestered in the lung tissue presented on Fig 2, Fig 7D-F, Fig 9D-F, Supplementary Fig 6A-C, Supplementary Fig 7A-D.

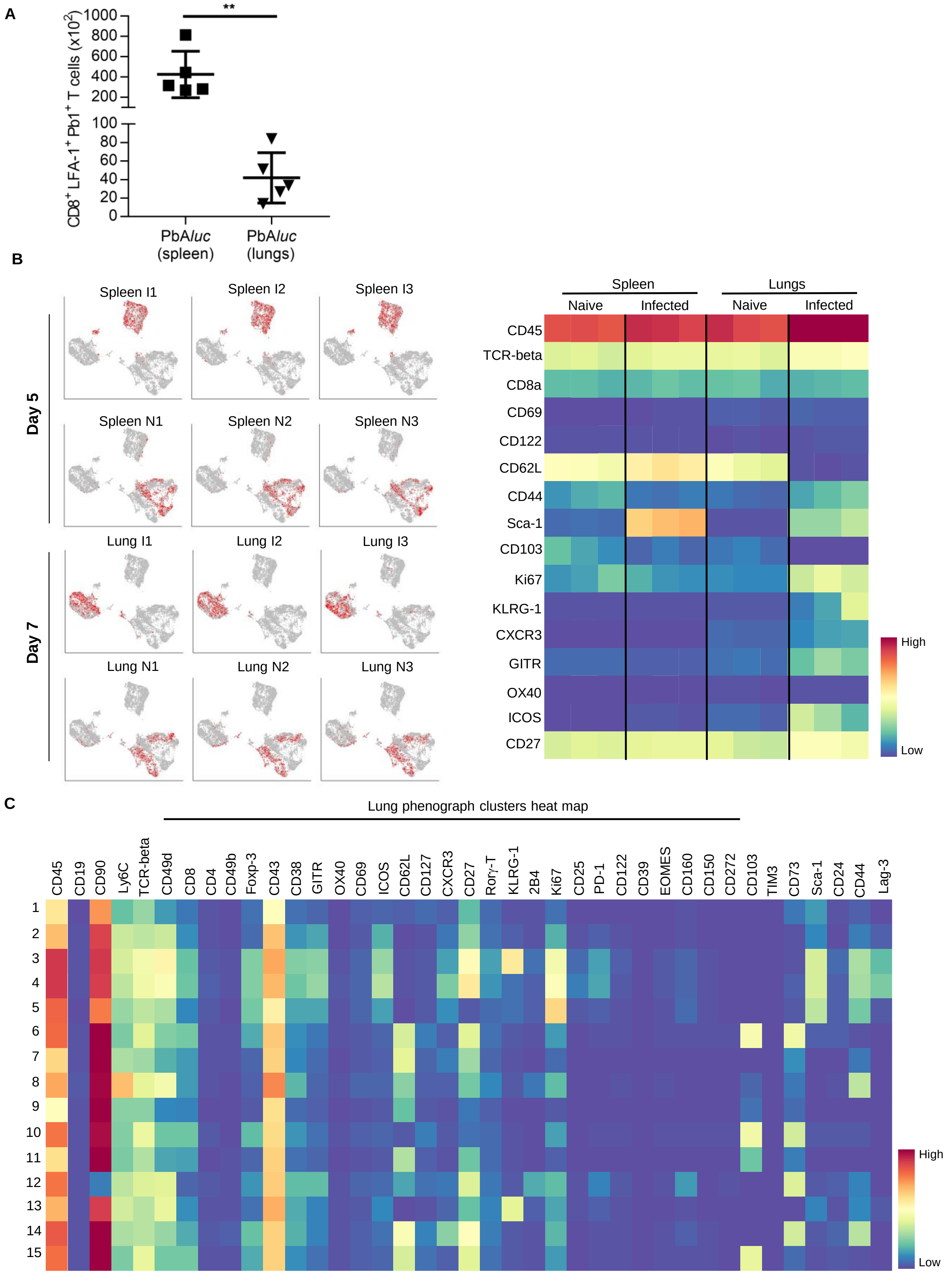


**Supplementary Figure 2. Quantification and characterization of CD8⁺ T cells present in the lungs and spleen.** (A)Total number of Pb1-specific CD8⁺ T cells/mL accumulated in the spleen and lungs of PbA*luc*-infected (n=5) mice at 5 dpi. The data represent the mean ± SD; **p<0.001, by Mann-Whitney test. (B) UMAP dimensionality reduction of CD8⁺ T cells isolated from C57BL/6 mice naïve (n=3) and infected spleen (n=3) and lungs (n=3) (5 and 7 dpi, respectively), color-coded by individual samples. The median expression intensities of effector and activation markers probed were plotted and summarized as heat map. (C) Median expression intensities of all phenotypic markers probed across individual lung samples were plotted and summarized as heat map.

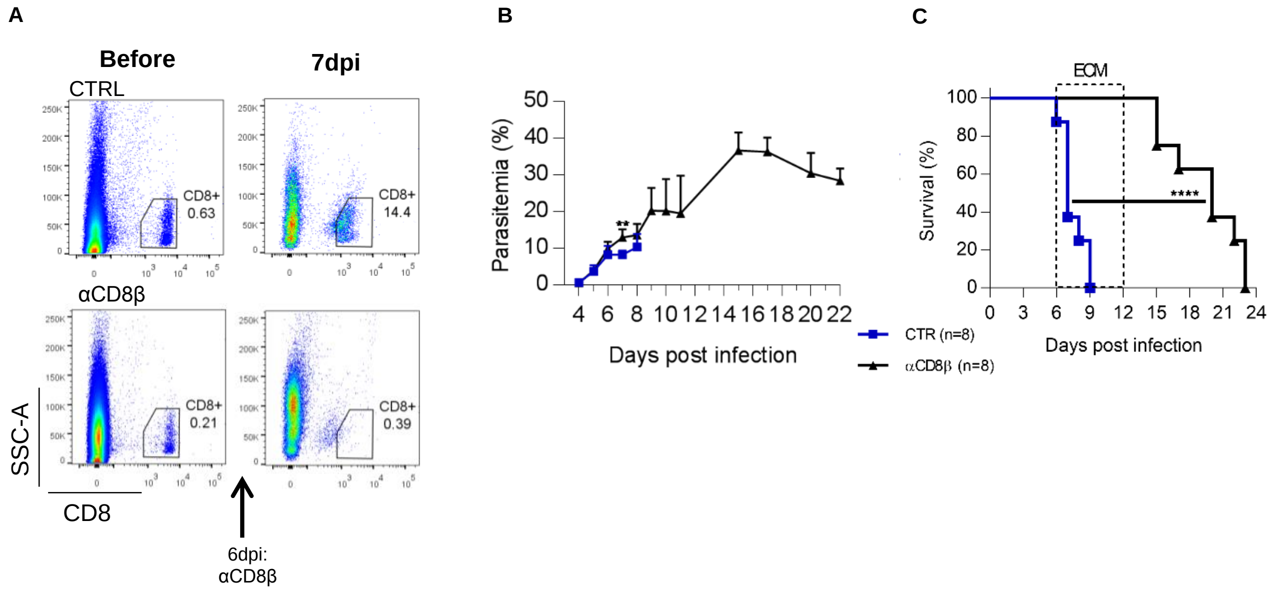


**Supplementary Figure 3. Anti-CD8β depletion efficiency.** (A) Flow cytometry dot plots depicting the CD8⁺T cell depletion efficiency in whole blood at 7 dpi in PbA*luc*-infected C57BL/6 mice before and after treatment with a single dose of 0.75mg of anti-mouse CD8β (αCD8β) administered on 6dpi. CTR represents PbA*luc*-infected C57BL/6 mice without treatment. (B) Peripheral parasitemia level and (C) survival curve of CTR (n=8) and αCD8β-treated (n=8) mice. The data represent the mean ± SD; **p<0.01 by Mann-Whitney test (B) and ****p<0.0001 by log-rank (Mantel-Cox) test (C).

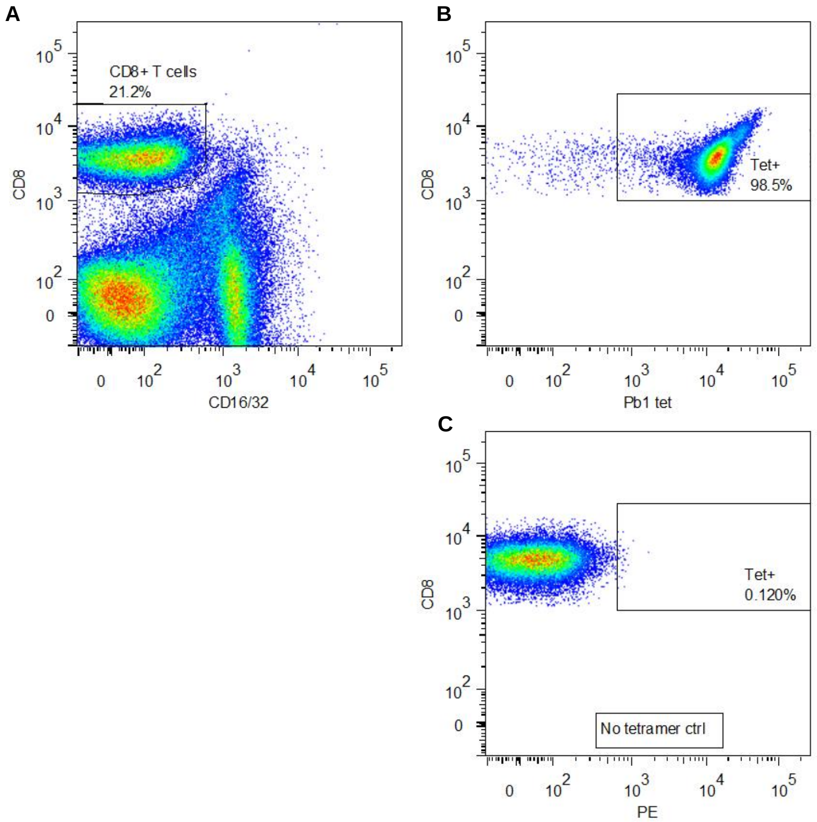


**Supplementary Figure 4. Pb1-speficifc CD8$^+$ T cells in transgenic BSL8.4 TCR mice.** CD8$^+$ T cells of BSL8.4 mice are Pb1-specific. Splenocytes were isolated from an 8.4a$^{+/-}$8.4b$^{+/-}$RAG1$^{-/-}$ mice and stained with αCD8, αCD16/32 and Pb1-tetramer (SQLLNAKYL-H-2D$^b$) and analysed by flow cytometry. (A) Gating of CD8$^+$ T cells. (B) Tetramer (Tet$^+$) staining on gated CD8$^+$ T cells. (C) Background signal when Pb1-tetramer was omitted.

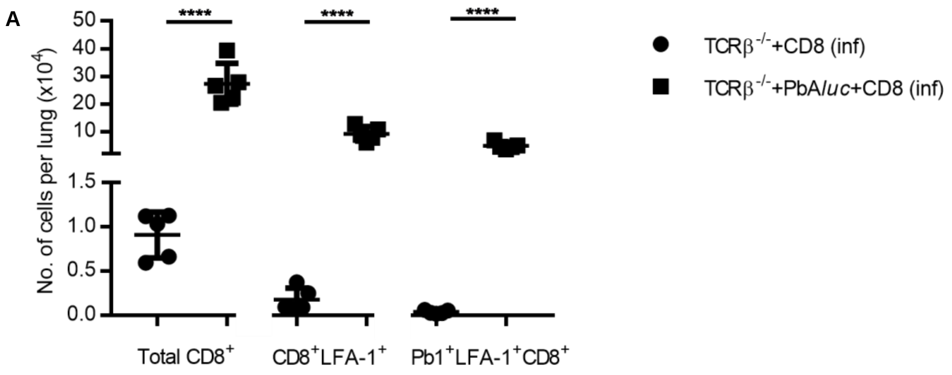


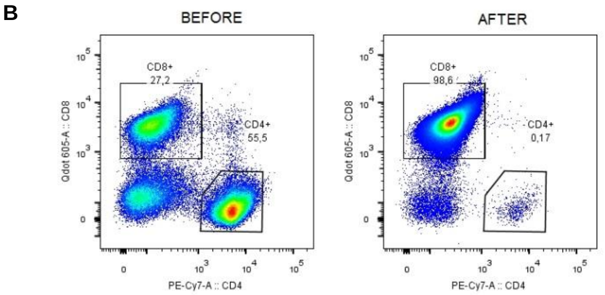


**Supplementary Figure 5. Donor CD8⁺ T cells from infected-C57BL/6 mice migrate into the lungs of TCRβ$^{-/-}$ only in the presence of PbA*luc* parasite.** (A) The number of total CD8$^+$, CD8$^+$ LFA-1$^+$ and Pb1-specific CD8$^+$ LFA-1$^+$ T cells in the lungs of recipient PbA*luc*-infected (or not) TCRβ$^{-/-}$ mice that were adoptively transferred with CD8$^+$ T cells from 7 dpi PbA*luc*-infected C57BL/6 donor (CD8 (inf)) mice. The data represent the mean ± SD; ****p<0.0001 by ANOVA with Bonferroni's post-test. (B) Flow cytometry dot plot showing the number of CD8$^+$ T cells isolated from splenocytes of donor C57BL/6 mice before and after enrichment using a CD8α$^+$T cell isolation kit.

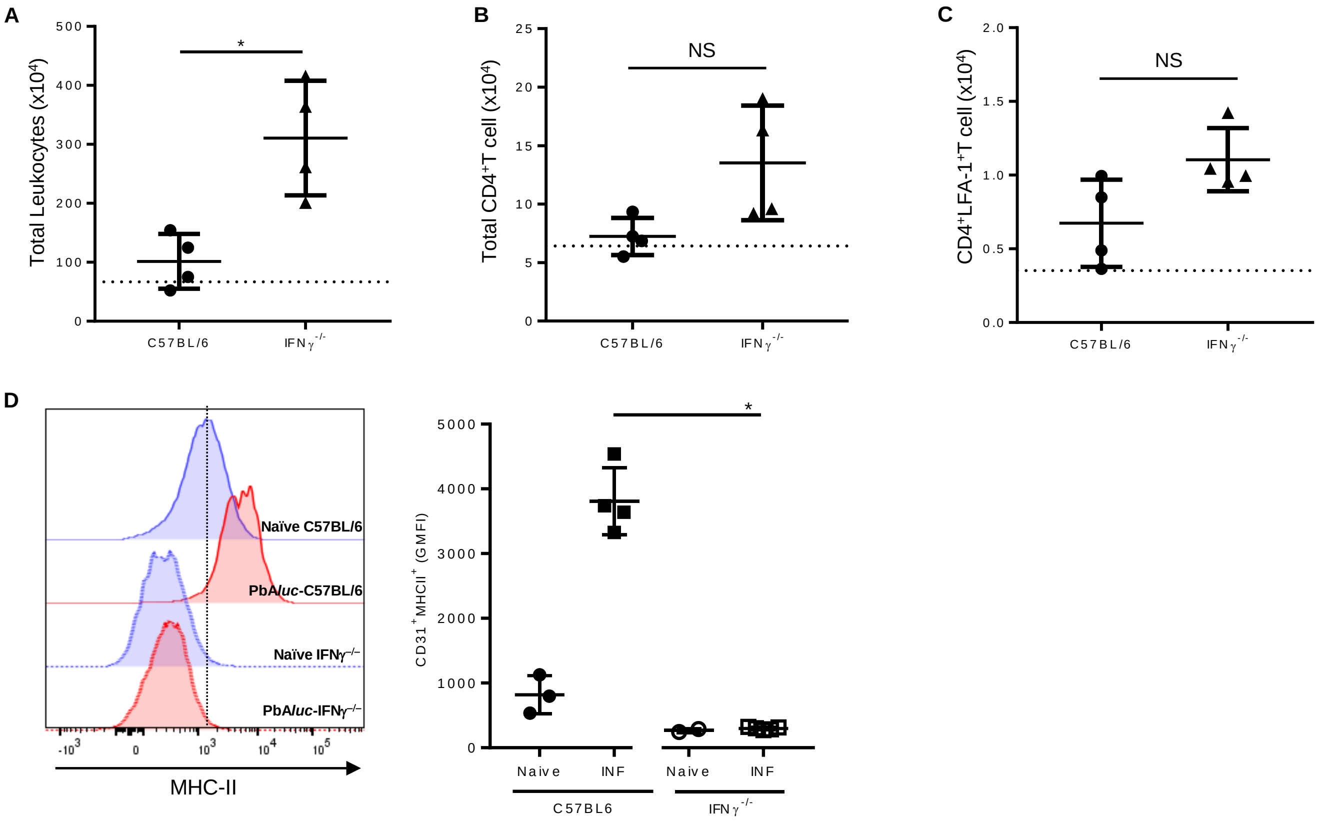


**Supplementary Figure 6. Absence of IFN-γ does not affect the migration of CD8⁺ T cells into infected mice.** (A) Leukocytes, (B) CD4⁺ T and (C) CD4⁺ LFA-1⁺ T cells in PbA*luc*-infected C57BL/6 (n=4) and IFN-$\gamma^{-/-}$ mice (n=4) at 7dpi. (D) Representative histograms of sorted lung endothelial cells (CD45⁻CD31⁺) from C57BL/6 naïve (n=3) and IFN-$\gamma^{-/-}$ naïve (n=3) mice (blue line), and PbA*luc*-infected C57BL/6 (n=4) and IFN-$\gamma^{-/-}$ (n=5) mice (red line) at 7 dpi that were stained for MHC-class II antigen presenting molecules (left). The graph represents the geometric mean fluorescence intensities (GMFI) of MHC-class II on CD31⁺ lung endothelial cells (right) from naïve and infected (INF) mice. The black dotted line in (A-C) represents the value of each respective cell population from naïve mice for quantification of immune-cell populations in the lungs. The data represent the mean ± SD; *p<0.05, Mann-Whitney test.

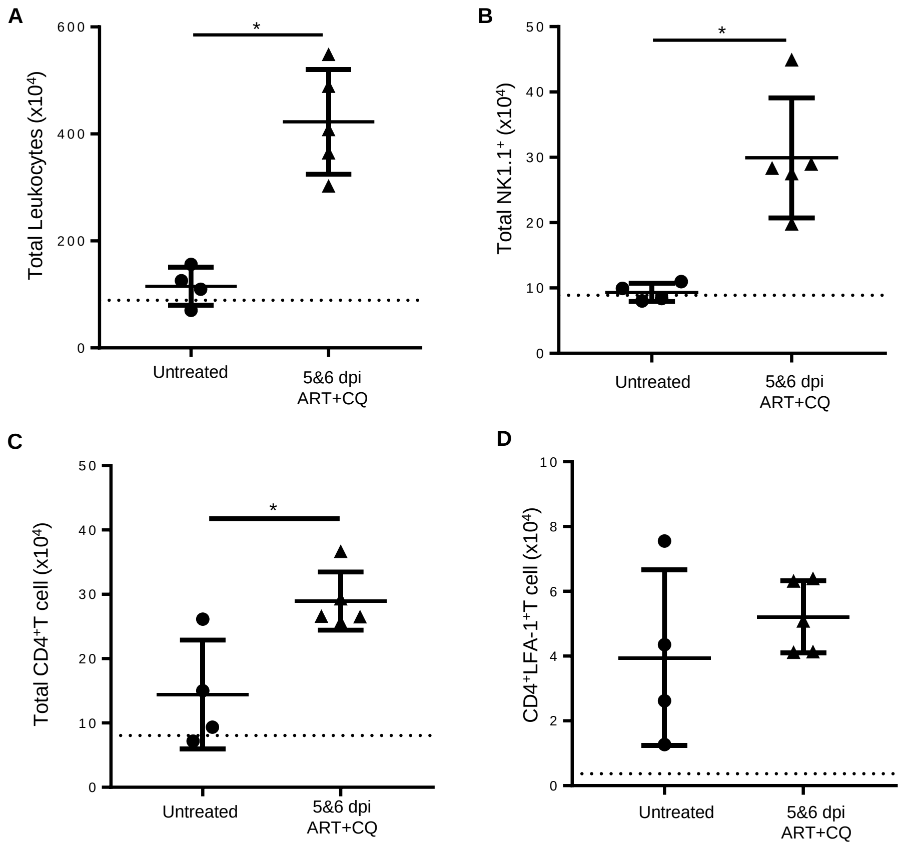


**Supplementary Figure 7. Antimalarial treatment enhances leukocytes migration into the lungs of PbA*luc*-infected mice.** Comparison between PbA*luc*-infected C57BL/6 mice (untreated) (n=4) and PbA*luc*-infected C57BL/6 mice that were treated with artesunate and chloroquine (ART+CQ) at 5 and 6 dpi (n=5) mice. Analyses were performed at 7 dpi. Total number of (A) leukocytes, (B) NK1.1$^+$ cells, (C) CD4$^+$ T and (D) CD4$^+$ LFA-1$^+$ T cells. The black dotted line in (A-D) represents the value of each respective cell population from naïve C57BL/6 mice for quantification of immune-cell populations in the lungs. The data represent the mean ± SD; *p<0.05, Mann-Whitney test.

**A**

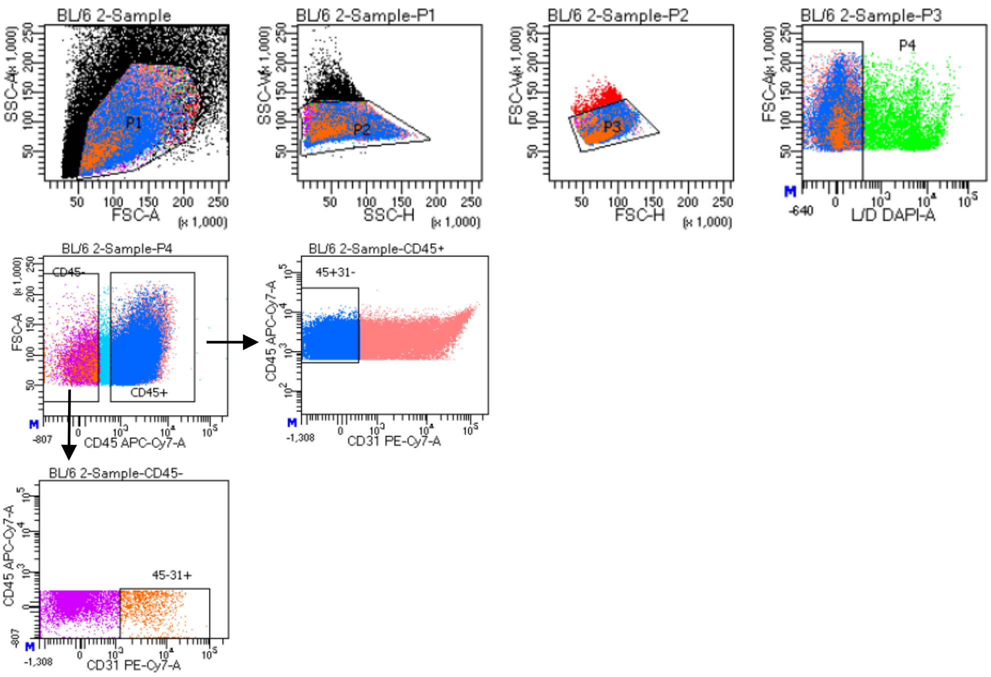


**B**

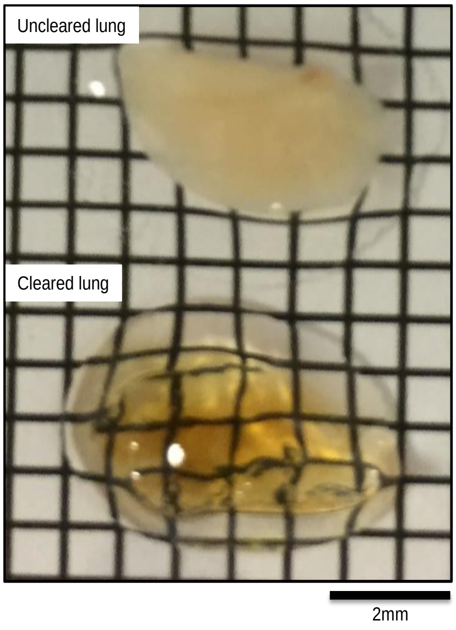


**Supplementary Figure 8.** (A) Sorting gating strategy of $CD45^+CD31^-$ leukocytes and $CD45^-CD31^+$ endothelial cells. The sorting was done on singlets, $DAPI^-CD45^+$ and $CD45^-$, followed by $CD45^+CD31^-$ and $CD45^-CD31^+$. Each sorted population was tested for Pb1 cross-presentation presented on Fig 6, Fig 7G and Fig 9G. L/D = live/dead. (B) Representative image of lung tissue before and after clearance with Benzyl Alcohol Benzyl Benzoate (BABB).
