# Supplementary material for: Lung endothelial cell antigen cross-presentation to CD8+T cells drives malaria-associated lung injury
Source: Nat Commun. 2019 Sep 18;10:4241. doi: 10.1038/s41467-019-12017-8 (PMC6751193; doi:10.1038/s41467-019-12017-8)
Supplement: Supplementary file 3 — Reporting Summary [file 41467_2019_12017_MOESM3_ESM.pdf]

nature research

Corresponding author(s): DBPR NCOMMS-19-03069A

Last updated by author(s): Jul 29, 2019

Reporting Summary

Nature Research wishes to improve the reproducibility of the work that we publish. This form provides structure for consistency and transparency in reporting. For further information on Nature Research policies, see Authors & Referees and the Editorial Policy Checklist.

Statistics

For all statistical analyses, confirm that the following items are present in the figure legend, table legend, main text, or Methods section.

n/a	Confirmed	
☐	☒	The exact sample size (*n*) for each experimental group/condition, given as a discrete number and unit of measurement
☐	☒	A statement on whether measurements were taken from distinct samples or whether the same sample was measured repeatedly
☐	☒	The statistical test(s) used AND whether they are one- or two-sided<br>*Only common tests should be described solely by name; describe more complex techniques in the Methods section.*
☒	☐	A description of all covariates tested
☐	☒	A description of any assumptions or corrections, such as tests of normality and adjustment for multiple comparisons
☐	☒	A full description of the statistical parameters including central tendency (e.g. means) or other basic estimates (e.g. regression coefficient) AND variation (e.g. standard deviation) or associated estimates of uncertainty (e.g. confidence intervals)
☐	☒	For null hypothesis testing, the test statistic (e.g. *F*, *t*, *r*) with confidence intervals, effect sizes, degrees of freedom and *P* value noted<br>*Give P values as exact values whenever suitable.*
☒	☐	For Bayesian analysis, information on the choice of priors and Markov chain Monte Carlo settings
☒	☐	For hierarchical and complex designs, identification of the appropriate level for tests and full reporting of outcomes
☒	☐	Estimates of effect sizes (e.g. Cohen's *d*, Pearson's *r*), indicating how they were calculated

Our web collection on statistics for biologists contains articles on many of the points above.

Software and code

Policy information about availability of computer code

Data collection	Flow cytometry data were collected using BD LSRFortessa™
Data analysis	• Flow cytometry data analysis was performed using FlowJo software version 10 (Tree Star) for Windows<br>• Statistical analysis between groups was performed with Graph pad prism version 7.03

For manuscripts utilizing custom algorithms or software that are central to the research but not yet described in published literature, software must be made available to editors/reviewers. We strongly encourage code deposition in a community repository (e.g. GitHub). See the Nature Research guidelines for submitting code & software for further information.

Data

Policy information about availability of data

All manuscripts must include a data availability statement. This statement should provide the following information, where applicable:

- Accession codes, unique identifiers, or web links for publicly available datasets
- A list of figures that have associated raw data
- A description of any restrictions on data availability

The source data underlying Figs 1a, 2a–d, 6d, h and 7c and Supplementary Figs 1a and 5d are provided as a Source Data file

Field-specific reporting

Please select the one below that is the best fit for your research. If you are not sure, read the appropriate sections before making your selection.

☒ Life sciences ☐ Behavioural & social sciences ☐ Ecological, evolutionary & environmental sciences

Life sciences study design

All studies must disclose on these points even when the disclosure is negative.

Sample size	No sample-size calculations were performed. Sample size was determined to be adequate based on the magnitude and consistency of measurable differences between groups.
Data exclusions	Samples were not excluded unless a severe anomaly was detected in the cell size gate during flow cytometry analysis or the mouse had a delay in peripheral parasitemia.
Replication	All data was successfully replicated at least two times.<br>How many times each experiment was performed and which statistical analysis was used is indicated in the figure legends.
Randomization	Samples were not randomized. Mice were age and sex matched and allocated to different groups.
Blinding	The investigators were not blinded to group allocation during data collection and analysis.

Reporting for specific materials, systems and methods

We require information from authors about some types of materials, experimental systems and methods used in many studies. Here, indicate whether each material, system or method listed is relevant to your study. If you are not sure if a list item applies to your research, read the appropriate section before selecting a response.

Materials & experimental systems

n/a	Involved in the study	
☐	☒	Antibodies
☐	☒	Eukaryotic cell lines
☒	☐	Palaeontology
☐	☒	Animals and other organisms
☒	☐	Human research participants
☒	☐	Clinical data

Methods

n/a	Involved in the study	
☒	☐	ChIP-seq
☐	☒	Flow cytometry
☐	☒	MRI-based neuroimaging

Antibodies

Antibodies used	Antibody are listed below as: target, clone, label, supplier, dilution<br>CD45 (30-F11), APC-Cy7, BD Pharmingen (1:250)<br>CD3e (145-2c11), BV421, Biolegend (1:100)<br>CD8 (53-6.7), BV605, Biolegend (1:200)<br>CD4 (RM4-5), PE-Cy7, eBioscience (1:200)<br>NK1.1 (Pk136), APC, eBioscience (1:100)<br>CXCR3 (CXCR3-173), PerCP-Cy5.5, eBioscience (1:100)<br>CD11a (LFA-1, M17/4), FITC, eBioscience (1:250)<br>IAb/IEb (MHC II, M5/114.15.2), Alexa700, eBioscience (1:200)<br>CD11b (M1/70), BV650, Biolegend (1:200)<br>CD11c (N418), PE-Cy7, eBioscience (1:200)<br>CD24 (M1/69), Pacblue, eBioscience (1:200)<br>CD64 (X54-5/7.1.1), PE, BD Pharmingen (1:100)<br>Ly6C (HK1.4), PerCP-Cy5.5, eBioscience (1:200)<br>F4/80 (BM8), Biotin, Biolegend (1:50)<br>FceR1 alpha (MAR1), APC, eBioscience (1:50)<br>BST2 (ebio927), FITC, eBioscience (1:200)<br>IFN-gama (XMG1.2), PerCP, eBioscience (1:50)<br>Granzyme B (NGZB), PE-Cy7, eBioscience (1:100)<br>CD31 (cl 390), PE-Cy7, Biolegend (1:50)<br>CD144 (VE-Cad, BV13), APC, eBioscience (1:100)<br>CD34 (RAM34), Alexa700, eBioscience (1:100)<br>L-selectin (CD62L, MEL-14), efluor450, eBioscience (1:100)<br>H-2Db (MHC I, KH95), FITC, Biolegend (1:200)<br>IAb/IEb (MHC II, M5/114.15.2), efluor450, eBioscience (1:200)<br>Streptavidin, PE-CF594, BD horizion, (1:200)
Validation	The monoclonal antibodies listed above are standard reagents used in the field and validated in the literature as cited on the manufacturers websites, as well as by the manufacturers data sheets themselves. The tetramers were validated in-house and

have been published previously.

Eukaryotic cell lines

Policy information about cell lines

Cell line source(s)	N/A
Authentication	N/A
Mycoplasma contamination	N/A
Commonly misidentified lines (See ICLAC register)	N/A

Animals and other organisms

Policy information about studies involving animals; ARRIVE guidelines recommended for reporting animal research

Laboratory animals	Mice used in the experiments were 6-7 weeks old male or female, all derived from a congenic C57BL/6J background. C57BL/6J, TCR KO, uGFP, IFN-gama KO and LR-BSL8.4 TCR transgenic mice were obtained from Biomedical Resource Centre (BRC)
Wild animals	Study did not involve wild animals.
Field-collected samples	Study did not involve samples collected from the field.
Ethics oversight	All protocols were approved by the BRC Institutional Animal Care and Use Committee (IACUC #181314) following the National Advisory Committee for Laboratory Animal Research (NACLAR) guidelines and Agri-Food and Veterinary Authority (AVA) rules.

Note that full information on the approval of the study protocol must also be provided in the manuscript.

Flow Cytometry

Plots

Confirm that:

- ☐ The axis labels state the marker and fluorochrome used (e.g. CD4-FITC).
- ☐ The axis scales are clearly visible. Include numbers along axes only for bottom left plot of group (a 'group' is an analysis of identical markers).
- ☒ All plots are contour plots with outliers or pseudocolor plots.
- ☒ A numerical value for number of cells or percentage (with statistics) is provided.

Methodology

Sample preparation	Samples were prepared as described in the methods section. Briefly, all mice tissues were harvested following perfusion. Single cell suspensions from lungs was obtained by enzymatic digestion with Collagenase D (Worthington, USA) and DNase I (RocheTM) as described in the methods section. All suspensions were ran through a 70 µm cell strainer. Erythrocytes were lysed using cold hypotonic solution (ACK). Before staining with specific antibodies, Live/Dead aqua was used to exclude dead cells.
Instrument	BD LSR Fortessa™
Software	FlowJo software version 10 (Tree Star) for Windows
Cell population abundance	N/A
Gating strategy	All gate strategies start by FSC vs SSC area, single cells by FSC height versus area, and live cells. Figure exemplifying the gating strategy is provided in the supplementary figures.

- ☒ Tick this box to confirm that a figure exemplifying the gating strategy is provided in the Supplementary Information.

Magnetic resonance imaging

Experimental design

Design type	T 2 weighted anatomical
Design specifications	Not applicable
Behavioral performance measures	Not applicable

Acquisition

Imaging type(s)	Anatomical
Field strength	9.4 Tesla
Sequence & imaging parameters	T2 weighted RARE imaging sequence. TR/TE = 1,200 ms/20 ms
Area of acquisition	Abdomen and thorax

Diffusion MRI ☐ Used ☒ Not used

Preprocessing

Preprocessing software	Bruker Paravision 6 and FIJI (Image J)
Normalization	Not applicable
Normalization template	Not applicable
Noise and artifact removal	Respiratory gating
Volume censoring	Not applicable

Statistical modeling & inference

Model type and settings	1 way ANOVA
Effect(s) tested	1 way ANOVA

Specify type of analysis: ☐ Whole brain ☒ ROI-based ☐ Both

Anatomical location(s): Lungs

Statistic type for inference (See Eklund et al. 2016)	Not applicable
Correction	None

Models & analysis

n/a	Involved in the study
☒	☐ Functional and/or effective connectivity
☒	☐ Graph analysis
☒	☐ Multivariate modeling or predictive analysis
