# Supplementary material for: Lung endothelial cell antigen cross-presentation to CD8+T cells drives malaria-associated lung injury
Source: Nat Commun. 2019 Sep 18;10:4241. doi: 10.1038/s41467-019-12017-8 (PMC6751193; doi:10.1038/s41467-019-12017-8)
Supplement: Supplementary file 4 — Description of Additional Supplementary Files [file 41467_2019_12017_MOESM4_ESM.pdf]

Description of Additional Supplementary Files

File Name: Supplementary Movie 1

Description: **CD8$^+$ T cells migrate in the lungs of PbAluc-infected TCRβ $^{-/-}$.** Light sheet imaging of cleared lungs of naïve TCRβ $^{-/-}$ mice, stained with anti-CD31, which indicates the vasculature (green). Movie length: 58 sec.

File Name: Supplementary Movie 2

Description: **CD8$^+$ T cells migrate in the lungs of PbAluc-infected TCRβ $^{-/-}$** . Light sheet imaging of cleared lungs of PbAluc-infected TCRβ $^{-/-}$ mice that have received CD8$^+$ T cells isolated from µGFP mice at 7 dpi, stained with anti-GFP, which indicates CD8$^+$ T cells (pink) and with anti-CD31, which indicates the vasculature (green). Movie length: 58 sec.
